# A Genome-Wide Association Study Identifies Susceptibility Variants for Type 2 Diabetes in Han Chinese

**DOI:** 10.1371/journal.pgen.1000847

**Published:** 2010-02-19

**Authors:** Fuu-Jen Tsai, Chi-Fan Yang, Ching-Chu Chen, Lee-Ming Chuang, Chieh-Hsiang Lu, Chwen-Tzuei Chang, Tzu-Yuan Wang, Rong-Hsing Chen, Chiung-Fang Shiu, Yi-Min Liu, Chih-Chun Chang, Pei Chen, Chien-Hsiun Chen, Cathy S. J. Fann, Yuan-Tsong Chen, Jer-Yuarn Wu

**Affiliations:** 1 School of Post-Baccalaureate Chinese Medicine, China Medical University, Taichung, Taiwan; 2 Department of Medical Genetics, Pediatrics and Medical Research, China Medical University Hospital, Taichung, Taiwan; 3 Department of Biotechnology and Bioinformatics, Asia University, Taichung, Taiwan; 4 Institute of Biomedical Sciences, Academia Sinica, Taipei, Taiwan; 5 National Genotyping Center, Academia Sinica, Taipei, Taiwan; 6 Division of Endocrinology and Metabolism, Department of Medicine, China Medical University Hospital, Taichung, Taiwan; 7 School of Chinese Medicine, College of Chinese Medicine, China Medical University, Taichung, Taiwan; 8 Department of Internal Medicine, National Taiwan University Hospital, Taipei, Taiwan; 9 Department of Internal Medicine, Endocrinology and Metabolism, Chia-Yi Christian Hospital, Chia-Yi, Taiwan; 10 Department of Pediatrics, Duke University Medical Center, Durham, North Carolina, United States of America; 11 Graduate Institute of Chinese Medical Science, China Medical University, Taichung, Taiwan; Queensland Institute of Medical Research, Australia

## Abstract

To investigate the underlying mechanisms of T2D pathogenesis, we looked for diabetes susceptibility genes that increase the risk of type 2 diabetes (T2D) in a Han Chinese population. A two-stage genome-wide association (GWA) study was conducted, in which 995 patients and 894 controls were genotyped using the Illumina HumanHap550-Duo BeadChip for the first genome scan stage. This was further replicated in 1,803 patients and 1,473 controls in stage 2. We found two loci not previously associated with diabetes susceptibility in and around the genes protein tyrosine phosphatase receptor type D (*PTPRD*) (*P* = 8.54×10^−10^; odds ratio [OR] = 1.57; 95% confidence interval [CI] = 1.36–1.82), and serine racemase (*SRR*) (*P* = 3.06×10^−9^; OR = 1.28; 95% CI = 1.18–1.39). We also confirmed that variants in *KCNQ1* were associated with T2D risk, with the strongest signal at rs2237895 (*P* = 9.65×10^−10^; OR = 1.29, 95% CI = 1.19–1.40). By identifying two novel genetic susceptibility loci in a Han Chinese population and confirming the involvement of *KCNQ1*, which was previously reported to be associated with T2D in Japanese and European descent populations, our results may lead to a better understanding of differences in the molecular pathogenesis of T2D among various populations.

## Author Summary

Type 2 diabetes (T2D) is a complex disease that involves many genes and environmental factors. Genome-wide and candidate-gene association studies have thus far identified at least 19 regions containing genes that may confer a risk for T2D. However, most of these studies were conducted with patients of European descent. We studied Chinese patients with T2D and identified two genes, PTPRD and SRR, that were not previously known to be involved in diabetes and are involved in biological pathways different from those implicated in T2D by previous association reports. PTPRD is a protein tyrosine phosphatase and may affect insulin signaling on its target cells. SRR encodes a serine racemase that synthesizes D-serine from L-serine. Both D-serine (coagonist) and the neurotransmitter glutamate bind to NMDA receptors and trigger excitatory neurotransmission in the brain. Glutamate signaling also regulates insulin and glucagon secretion in pancreatic islets. Thus, SRR and D-serine, in addition to regulating insulin and glucagon secretion, may play a role in the etiology of T2D. Our study suggests that, in different patient populations, different genes may confer risks for diabetes. Our findings may lead to a better understanding of the molecular pathogenesis of T2D.

## Introduction

Type 2 diabetes (T2D) affects at least 6% of the world's population; the worldwide prevalence is expected to double by 2025 [1]. T2D is a complex disorder that is characterized by hyperglycemia, which results from impaired pancreatic β cell function, decreased insulin action at target tissues, and increased glucose output by the liver [2]. Both genetic and environmental factors contribute to the pathogenesis of T2D. The disease is considered to be a polygenic disorder in which each genetic variant confers a partial and additive effect. Only 5%–10% of T2D cases are due to single gene defects; these include maturity-onset diabetes of the young (MODY), insulin resistance syndromes, mitochondrial diabetes, and neonatal diabetes [3]–[5]. Inherited variations have been identified from studies of monogenic diabetes, and have provided insights into β cell physiology, insulin release, and the action of insulin on target cells [6].

Much effort has been devoted to finding common T2D genes, including genome-wide linkage, candidate-gene, and genome-wide association studies (GWAS). Whole-genome linkage scans have identified chromosomal regions linked to T2D; however, with the exception of regions 1q [7]–[13] and 20q, which have been repeatedly mapped, linkage results vary from study to study [14]–[19]. Candidate-gene studies have provided strong evidence that common variants in the peroxisome proliferator-activated receptor-r (*PPARG*) [20], potassium inwardly-rectifying channel J11 (*KCNJ11*) [21]–[23], transcription factor 2 isoform b (*TCF2*) [24],[25], and Wolfram syndrome 1 (*WFS1*) [26] genes are associated with T2D. These genes all have strong biological links to diabetes, and rare, severe mutations cause monogenic diabetes. GWAS have accelerated the identification of T2D susceptibility genes, expanding the list from three in 2006 to over 20 genes in 2009. There are now at least 19 loci containing genes that increase risk of T2D, including *PPARG* [27], *KCNJ11* [27], *KCNQ1* [28],[29], *CDKAL1* [27],[29]–[33], *CDKN2A-2B* [27],[32],[33], *CDC123-CAMK1D* [34], *MTNR1B* [35]–[37], *TCF7L2* [31],[38],[39], *TCF2* (*HNF1B*), *HHEX-KIF11-IDE* [27],[32],[33],[38], *JAZF1* [34], *IGF2BP2* [27],[29],[32], *SLC30A8* [27],[32],[33],[38], *THADA* [34], *ADAMTS9* [34], *WFS1* [26], *FTO* [27],[31], *NOTCH2* [34], and *TSPAN8* [34]. Variants in these genes have been identified almost exclusively in populations of European descent, except for *KCNQ1*; individually, these variants confer a modest risk (odds ratio [OR] = 1.1–1.25) of developing T2D. *KCNQ1* was identified as a T2D susceptibility gene in three GWA scans in Japanese individuals, highlighting the need to extend large-scale association efforts to different populations, such as Asian populations [28],[29],[40]. The association of other previously reported loci (*CDKAL1*, *CDKN2A-2B*, *IGF2BP2*, *TCF7L2*, *SLC30A8*, *HHEX*, and *KCNJ11*) with T2D were also replicated in the Japanese population [29],[40],[41].

To date, a GWA scan for T2D has not been conducted in the Han Chinese population, although the association of some known loci have been confirmed, including *KCNQ1* and *CDKAL1*, *CDKN2A-2B*, *MTNR1B*, *TCF7L2*, *HNF1β*, and *KCNJ11* [42]–[47]. Therefore, we conducted a two-stage GWA scan for T2D in a Han Chinese population residing in Taiwan. There were a total of 2,798 cases and 2,367 normal controls (995 cases and 894 controls in stage 1, 1,803 cases and 1,473 controls in stage 2). Our accomplished objective was to identify new diabetes susceptibility loci that were associated with increased risk of T2D in a Han Chinese population.

## Results

### Association analysis

**Figure 1:**
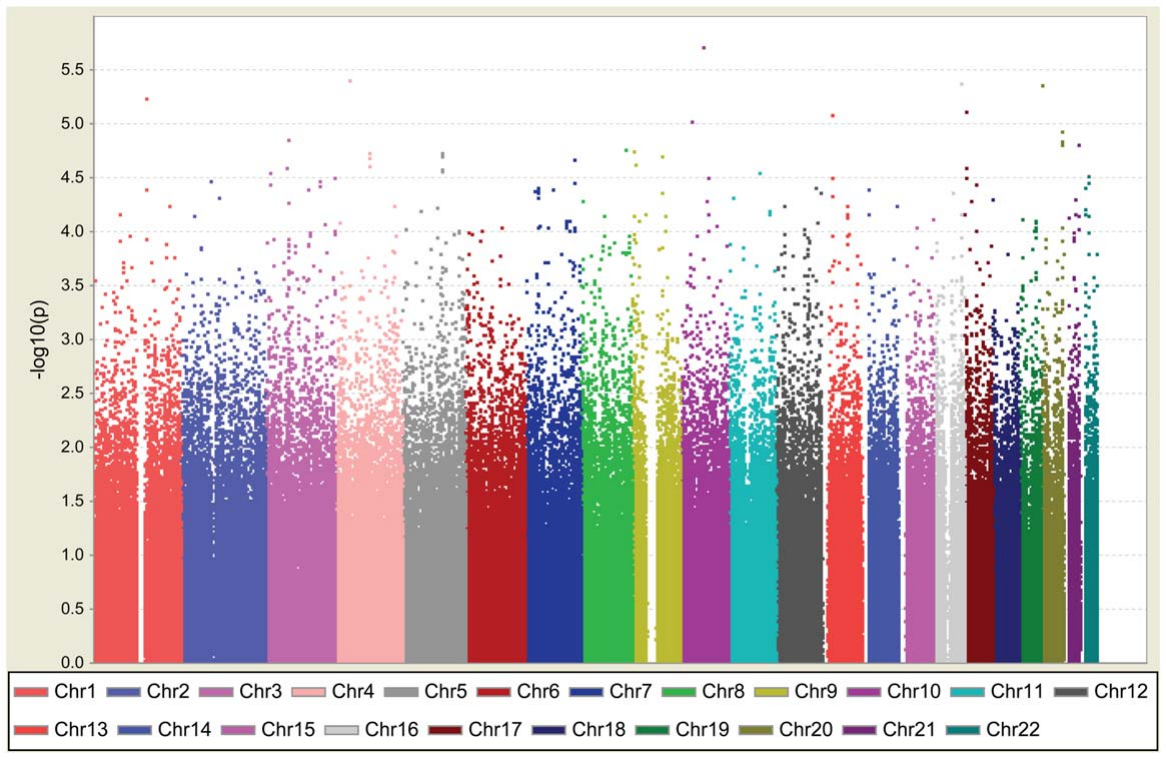
Graphical summary of T2D GWAS in a Han Chinese population. T2D association was determined for SNPs on the Illumina HumanHap550K-Duo chip. The y-axis represents the −log_10_ *P* value and the x-axis represents each of the 516,212 SNPs used in the primary scan of 995 T2D cases and 894 controls.

We conducted a two-stage GWAS to identify genetic variants for T2D in the Han-Chinese residing in Taiwan. In the first stage, an exploratory genome-wide scan, we genotyped 995 T2D cases and 894 population controls using the Illumina Hap550duov3 chip (Figure 1 and Table S1). For each sample genotyped in this study, the average call rate was 99.92±0.12%. After applying stringent quality control criteria, high-quality genotypes for 516,737 SNPs (92.24%) were obtained, with an average call rate of 99.92±0.24% (Table S2). The results of principal component analysis in stage 1 revealed no evidence for population stratification between T2D cases and controls (*P* = 0.111, Fst statistics between populations <0.001) (Text S1; Figure S1). Multidimensional scaling analysis using PLINK [48] produced similar results (Text S1; Figure S2). Furthermore, genomic control (GC) with a variance inflation factor λ = 1.078 (trend test) did not substantially change the results of this GWAS (Table S3).

We selected eight SNPs in seven regions: rs9985652 and rs2044844 on 4p13, rs7192960 on 16q23.1, rs7361808 on 20p13, rs1751960 on 10q11.23, rs4845624 on 1q21.3, rs391300 on 17p13.3, and rs648538 on 13q12.3. These SNPs had association *P* values of <10^−5^ at stage 1 with any of the genotype, allele, trend, dominant, and recessive models for subsequent cross-platform validation using Sequenom (Table 1; Table S3). For SNPs with weaker associations (*P* value between 10^−4^ and 10^−5^), we searched for novel susceptibility candidates for T2D as implicated by (1) gene function identified by a bioinformatics approach and (2) an animal model showing defects in glucose homeostasis caused by genes within the same subfamily. Therefore, we selected SNP rs17584499 (*P* = 2.4×10^−5^ under best model) for further investigation. rs17584499 lies within protein tyrosine phosphatase receptor type D (*PTPRD*). We hypothesized that PTPRD might play a role in the regulation of insulin signaling, because its subfamily members leukocyte common antigen-related (*LAR*) and protein tyrosine phosphatase sigma (*PTPRS*) exhibit defects in glucose homeostasis and insulin sensitivity in knockout and/or transgenic mice [49]–[51].

**Table 1:** Association results for Type 2 diabetes in Han Chinese.

									Joint anaysis of stage1+2			
SNP^a^	Chr.	Nearest gene(s)	Risk allele	stage	RAF (T2D)	RAF (NC)	OR (95% CI)	*P* value (trend)	OR (95% CI)	*P* value (trend)	*P* value (perm)	*P* value (Fisher)
rs9985652	4	ATP8A1	A	1	0.54	0.48	1.25 (1.10–1.42)	9.30×10^−4^				
		GRXCR1		2	0.51	0.52	0.95 (0.86–1.05)	0.316	1.05 (0.97–1.14)	0.193	0.156	0.003
rs2044844	4	ATP8A1	C	1	0.53	0.48	1.24 (1.09–1.41)	9.30×10^−4^				
		GRXCR1		2	0.51	0.52	0.96 (0.87–1.06)	0.406	1.06 (0.98–1.14)	0.17	0.141	0.003
rs7192960	16	MAF	C	1	0.75	0.68	1.39 (1.20–1.61)	7.66×10^−6^				
		WWOX		2	0.73	0.70	1.12 (1.01–1.25)	0.037	1.21 (1.11–1.33)	1.33×10^−5^	6.82×10^−6^	4.61×10^−6^
rs7361808	20	SIRPA	G	1	0.09	0.06	1.60 (1.24–2.05)	2.30×10^−4^				
				2	0.06	0.06	1.07 (0.87–1.31)	0.52	1.25 (1.07–1.47)	0.005	0.005	0.001
rs1751960	10	LYZL1	G	1	0.53	0.46	1.34(1.18–1.53)	1.13×10^−5^				
		SVIL		2	0.50	0.49	1.05(0.95–1.16)	0.317	1.15(1.06–1.24)	3.78×10^−4^	4.05×10^−4^	4.85×10^−5^
rs4845624	1	RORC	A	1	0.65	0.58	1.36 (1.19–1.56)	5.85×10^−6^				
		TMEM5		2	0.61	0.63	0.91 (0.83–1.01)	0.07	1.05 (0.97–1.14)	0.205	0.203	6.46×10^−6^
**rs391300**	17	SRR	G	1	0.69	0.63	1.31 (1.14–1.50)	9.00×10^−5^				
				2	0.68	0.62	1.26 (1.14–1.40)	6.55×10^−6^	**1.28 (1.18–1.39)**	**3.06×10^−9^**	**1.00×10^−8^**	**5.52×10^−8^** *
**rs4523957^b^**	17	SRR	T	1	0.71	0.65	1.30 (1.12–1.49)	3.24×10^−4^				
				2	0.68	0.63	1.26 (1.14–1.40)	8.22×10^−6^	**1.27 (1.17–1.38)**	**1.44×10^−8^**	**3.00×10^−8^**	**1.31×10^−8^** *
rs648538	13	KATNAL1	G	1	0.67	0.61	1.28 (1.12–1.47)	2.92×10^−4^				
				2	0.65	0.66	0.96 (0.86–1.06)	0.407	1.07(0.98–1.16)	0.116	0.125	0.001
**rs17584499**	9	PTPRD	T	1	0.11	0.07	1.55 (1.23–1.94)	1.41×10^−4^				
				2	0.09	0.06	1.61 (1.33–1.95)	9.15×10^−7^	**1.57 (1.36–1.82)**	**8.54×10^−10^**	**1.00×10^−8^**	**3.07×10^−9^** *
**rs231361**	11	KCNQ1	T	1	0.85	0.81	1.39 (1.17–1.64)	1.49×10^−4^				
				2	0.83	0.79	1.26 (1.11–1.44)	2.85×10^−4^	**1.30 (1.18–1.44)**	**2.89×10^−7^**	**3.10×10^−7^**	**7.64×10^−7^** *
**rs231359^b^**	11	KCNQ1	A	1	0.85	0.81	1.36 (1.14–1.61)	4.56×10^−4^				
				2	0.84	0.80	1.32 (1.17–1.51)	1.68×10^−5^	**1.33 (1.20–1.48)**	**3.43×10^−8^**	**5.00×10^−8^**	**1.51×10^−7^** *
**rs2237895^c^**	11	KCNQ1	C	1	0.40	0.35	1.28 (1.12–1.46)	2.92×10^−4^				
				2	0.39	0.33	1.30 (1.17–1.45)	6.46×10^−7^	**1.29 (1.19–1.40)**	**9.65×10^−10^**	**1.00×10^−8^**	**4.42×10^−9^** *

**^a^**SNPs are arranged in order of decreasing *P* value under best statistical model in Stage 1.

**^b^**Neighboring SNPs also significantly associated with T2D.

**^c^**Previously reported SNP associated with T2D ^28, 29^ and validated in our study.

*Empirical *P* value <10^6^ after 10^8^ permutations.

Stage 1(Genome scan) included 995 cases and 894 controls. Stage 2 (replication stage) included 1,803 cases and 1,473 controls. Alleles were indexed to the forward strand of NCBI Build 36. *P* value (trend), *P* value (PC), *P* value (permutation), and *P* value (meta) represent *P* values of trend test, principal component analysis using EIGENSTRAT, permutation, and meta-analysis using Fisher's method, respectively.

Risk allele, allele with higher frequency in cases compared to controls; RAF (T2D) and RAF (NC), risk allele frequencies in cases and controls, respectively; and OR, odds ratio for risk allele.

We also evaluated the most significant SNP (rs231361) within *KCNQ1*, which was previously reported to be a diabetes susceptibility gene in a Japanese population, as well as in populations of Korean, Chinese, and European ancestry [28],[29]. Together, these ten SNPs—the 8 SNPs with association *p*<10^−5^, rs17584499, and rs231361—were cross-platform validated and yielded consistent results using both Illumina and Sequenom. The concordance rate for stage 1 samples typed on the Illumina and Sequenom platforms was 99.1%±0.84% (Table S4).

**Figure 2:**
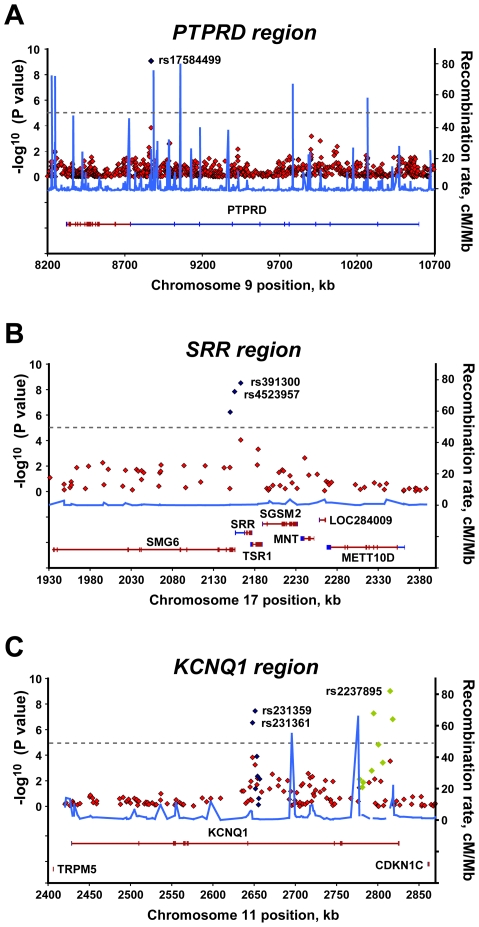
Regional plots of three significant associations. For each of the (A) *PTPRD*, (B) *SRR*, and (C) *KCNQ1* regions, the −log_10_ *P* values for the trend test from the primary scan were plotted as a function of genomic position (NCBI Build 36). The SNPs with the strongest signal and neighboring genotyped SNPs in the joint analysis are denoted by blue diamonds. Green diamonds in the *KCNQ1* region (C) represent reported T2D–associated SNPs genotyped in all samples of joint analysis. Estimated recombination rates (right y-axis) based on the Chinese HapMap population was plotted to reflect the local LD structure around the significant SNPs. Gene annotations were taken from NCBI.

We took these ten SNPs and an additional 29 neighboring SNPs within the linkage disequilibrium (LD) block forward to replicate in 3,803 additional samples (stage 2; 1,803 cases and 1,473 controls). The average call rate for each sample was 96.13%±4.66%. After applying stringent quality control criteria, high-quality genotypes for 35 SNPs (89.7%) were obtained, with an average call rate of 98.96%±0.24% (Table S2). Of the ten SNPs selected in stage 1, only three SNPs still showed a strong association in the stage 2 analysis: rs17584499 in *PTPRD* at 9p24.1-p23, rs231359 in *KCNQ1* at 11p15.5, and rs391300 in serine racemase (*SRR*) at 17p13.3 (Table 1). We were unable to replicate the association between T2D and the remaining seven SNPs in *ATP8A1/GRXCR1*, *MAF/WWOX*, *SIRPA*, *LYZL1/SVIL*, *RORC/TMEM5*, and *KATNAL1* in the stage 2 analysis (Table 1). Joint analysis of stage 1 and stage 2 data revealed consistent results with stage 2. The most significant associations were found for rs391300, rs17584499, and rs231359 (Table 1; Figure 2). These associations remained significant after calculating *P* values using 10^8^ permutations of the disease state labels. Joint association analysis was performed with all of the 2,798 T2D cases and 2,367 controls; this could achieve a power of 0.85 to detect a disease allele with a frequency of 0.15 and an OR of 1.5, assuming a disease prevalence of 0.06, at a significant level of 0.05 (Table S5).

### Identification of two novel T2D loci and confirmation of *KCNQ1* association

Two previously unknown loci were detected in our joint analysis of GWAS data. The strongest new association signal was found for rs17584499 in intron 10 of *PTPRD* (*P* = 8.54×10^−10^ [trend test]; allelic OR = 1.57, 95% confidence interval [CI] = 1.36–1.82) (Table 1; Figure 2). The second strongest signal was found with rs391300 (*P* = 3.06×10^−9^ [trend test]; OR = 1.28, 95% CI = 1.18–1.39). The nearby SNP rs4523957 also demonstrated a significant association (*P* = 1.44×10^−8^; OR = 1.27, 95% CI = 1.17–1.38). SNPs rs391300 and rs4523957 were in tight LD with one another (*r* ^2^ = 0.942 in HapMap HCB), and were located within the serine racemase gene (*SRR*).

SNP rs231361, located in intron 11 of *KCNQ1*, had a less significant association with T2D, and was selected in stage 1 (*P* = 1.49×10^−4^ [trend test]; OR = 1.39, 95% CI = 1.17–1.64) (Table 1). We further genotyped eight additional SNPs within the same LD block from the HapMap Asian group data: rs231359 yielded a *P* value of 4.56×10^−4^ with a trend test (OR = 1.36, 95% CI = 1.14–1.61) (Figure 2). rs231361 and rs231359 were in strong LD with one another (*r* ^2^ = 1 in HapMap HCB), and were located approximately 164 kb upstream of SNP rs2237897, which was previously reported to be significantly associated with T2D in a Japanese population [28],[29]. We took rs231361, rs231359, and neighboring SNPs within the LD block forward to replicate in stage 2. Joint analysis of stage 1 and stage 2 data revealed that rs231359 had an even stronger association with T2D than did rs231361 (rs231359: *P* = 3.43×10^−8^, OR = 1.33, 95% CI = 1.2–1.48; rs231361: *P* = 2.89×10^−7^, OR = 1.3, 95% CI = 1.18–1.44).

Additional SNPs that were reported to be significantly associated with T2D in a Japanese population were further genotyped [28],[29]. The average call rate for each sample was 99.12%±7.21%. After applying stringent quality control criteria, we obtained high-quality genotypes with an average call rate of 99.16%±0.18% (Table S2). SNP rs2237895 showed the strongest association with T2D of all the genotyped SNPs in *KCNQ1* (*P* = 9.65×10^−10^; OR = 1.29, 95% CI = 1.19–1.40) (Figure 2 and Figure S3; Table S6). Conditioning on the rs2237895, the statistical significance of rs231361 (or rs231359) disappeared. It seems the same underlying biological effect between the 2 SNPs (Table S7).

Subsequently, we sequenced all of the exons, intron–exon boundaries, and up to 1.2 kb of the promoter region of the *KCNQ1* gene in 50 individuals with T2D, and identified 42 polymorphic variations, including one nonsynonymous P448R polymorphism and two novel SNPs with minor allele frequency >0.03. We then genotyped the two novel SNPs and one nonsynonymous polymorphism; however, none of these SNPs showed an association with T2D (Table S6).

## Discussion

Our GWAS for T2D in a Han Chinese population found two previously unreported susceptibility genes. All of the significant variants detected in our study showed modest effects, with an OR between 1.21 and 1.57. Two loci with less-significant associations in our primary scan (stage 1), *PTPRD* and *KCNQ1*, were selected for further replication; both showed compelling evidence of association in joint analysis. The susceptibility loci we identified in this study need to be further replicated in additional populations. Of the 18 loci previously reported to be associated with T2D (with the exception of *KCNQ1*), none of the *P* values for any of the SNPs within or near the genes reached 10^−5^ using allele, genotype, trend, dominant, or recessive models (Table S8; Figure S4). Three SNPs within *CDKAL1*, *JAZF1*, and *HNF1B* had the lowest *P* values, ranging from 5×10^−4^ to 10^−5^, among the 18 known loci (Table S8). No significant associations were found within these regions in our Han Chinese population.

The strongest new signal was observed for rs17584499 in *PTPRD*. The overall Fst among 11 HapMap groups for rs17584499 was estimated to be 0.068 [52], which indicated a significant difference in allele frequencies among the populations (*P*<0.0001, chi-square test ) (Table S9). *PTPRD* is widely expressed in tissues, including skeletal muscle and pancreas, and is expressed highest in the brain. *PTPRD*-deficient mice exhibit impaired learning and memory, early growth retardation, neonatal mortality, and posture and motor defects [53]. Multiple mRNA isoforms are expressed by alternative splicing and/or alternative transcription start sites in a developmental and tissue-specific manner [54],[55]. *PTPRD* belongs to the receptor type IIA (R2A) subfamily of protein tyrosine phosphatases (PTPs). The R2A PTP subfamily comprises *LAR*, *PTPRS*, and *PTPRD*. The R2A family has been implicated in neural development, cancer, and diabetes [56]. Although the complex phenotype including neurological defects seen in knockout mice could obscure the roles of these genes in glucose homeostasis, *LAR*- and *PTPRS*-deficient mice were demonstrated to have altered glucose homeostasis and insulin sensitivity [49]–[51]. Transgenic mice overexpressing *LAR* in skeletal muscle show whole-body insulin resistance [57]. Because R2A subfamily members are structurally very similar [54], *PTPRD* could play a role in T2D pathogenesis and should be further characterized.

The second new association locus was found for rs391300 and rs4523957 in the biologically plausible candidate gene *SRR*. *SRR* encodes a serine racemase that synthesizes D-serine from L-serine [58],[59]. D-serine is a physiological co-agonist of the N-methyl D-aspartate (NMDA) class of glutamate receptors, the major excitatory neurotransmitter receptors mediating synaptic neurotransmission in the brain [60],[61]. NMDA receptor activation requires binding of glutamate and D-serine, which plays a neuromodulatory role in NMDA receptor transmission, synaptic plasticity, cell migration, and neurotoxicity [62]. D-serine and SRR are also present in the pancreas [63]. Glutamate signaling functions in peripheral tissues, including the pancreas, and positively modulates secretion of both glucagon and insulin in pancreatic islets [64]–[66]. The nearby SNP rs216193 also showed significant association (*P* = 2.49×10^−6^); this SNP resides 3.8 kb upstream from *SRR*, within Smg-6 homolog, nonsense mediated mRNA decay factor (*C. elegans*) (*SMG6*). rs216193 was in tight LD with rs391300 (*r* ^2^ = 0.942 in HapMap HCB). Based on their biological functions and the association results, neither *SMG6* nor any of the nearby genes *TSR1*, *SGSM2*, *MNT*, and *METT10D* were compelling candidates for association withT2D. However, *SRR* was significantly associated with T2D; thus, we suggest that dysregulation of D-serine could alter glutamate signaling and affect insulin or glucagon secretion in T2D pathogenesis.

rs7192960 also had a suggestive association with T2D (*P* = 1.32×10^−5^; OR = 1.21, 95% CI = 1.11–1.33). This SNP which lies approximately 211 kb downstream of v-maf musculoaponeurotic fibrosarcoma oncogene homolog (avian) (*MAF*) and 170 kb downstream of WW domain containing oxidoreductase (*WWOX*). *WWOX* is a tumor suppressor gene that spans the second most common human fragile site FRA16D [67],[68], and is disrupted in many tumors, including pancreatic carcinoma [67], [69]–[73]. *MAF* encodes the transcription factor c-Maf, a member of the Maf family of basic-Zip (bZip) transcription factors. c-Maf is involved in development and differentiation of the lens [74],[75], kidney [76], immune system [77], adipose tissue [78], and pancreas [79]. It is expressed in α cells of the pancreatic islets [80], and is a strong transactivator of the glucagon promoter that regulates glucagon gene expression [80],[81]. c-Maf is also associated with early-onset and morbid adult obesity [82].

Our GWAS revealed that *KCNQ1*, which was previously reported to be associated with T2D in several populations, was also associated with T2D in a Han Chinese population residing in Taiwan. *KCNQ1* encodes the pore-forming α subunit of a voltage-gated K^+^ channel (KvLQT1), which is involved in repolarization of the action potential in cardiac muscle [83],[84]. Mutations in *KCNQ1* cause long QT syndrome [85],[86] and familial atrial fibrillation [87]. *KCNQ1* is widely expressed, including in the heart, brain, kidney, liver, intestine, and pancreas [88]–[90]. It is also expressed in pancreatic islets, and blockade of the KvLQT1 channel stimulates insulin secretion in insulin-secreting INS-1 cells [91]. *KCNQ1* knockout mice have cardiac dysfunctions [88],[92] and enhanced systemic insulin sensitivity [93]. In our study, variants in the coding region did not show an association with T2D. The functional variant(s) could be located in the regulatory element of *KCNQ1*, rather than in the coding region. We did not find an association between either *CDKAL1* or *IGF2BP2* and T2D, in contrast with the results described in a previous study [29], nor did we find T2D associated with various other genes identified in populations of European descent.

In conclusion, we identified two previously unknown loci that are associated with T2D in a Han Chinese population, and confirmed the reported association of *KCNQ1* with T2D. The novel T2D risk loci may involve genes that are implicated in insulin sensitivity and control of glucagon and insulin secretion: *PTPRD* may participate in the regulation of insulin action on its target cells, while *SRR* variants may alter glutamate signaling in the pancreas, thus regulating insulin and/or glucagon secretion. Our study suggests that in different patient populations, different genes may confer risks for diabetes, which may lead to a better understanding of the molecular pathogenesis of T2D.

## Materials and Methods

### Ethical statement

The study was approved by the institutional review board and the ethics committee of each institution. Written informed consent was obtained from each participant in accordance with institutional requirements and the Declaration of Helsinki Principles.

### Subject participants

A total of 2,798 unrelated individuals with T2D, age >20 years, were recruited from China Medical University Hospital (CMUH), Taichung, Taiwan; Chia-Yi Christian Hospital (CYCH), Chia-Yi, Taiwan; and National Taiwan University Hospital (NTU), Taipei, Taiwan. All of the T2D cases were diagnosed according to medical records and fasting plasma glucose levels using American Diabetic Association Criteria. Subjects with type 1 diabetes, gestational diabetes, and maturity-onset diabetes of the young (MODY) were excluded from this study. For the two-stage GWAS, we genotyped 995 T2D cases and 894 controls in the first exploratory genome-wide scan (stage 1). In the replication stage (stage 2), we genotyped selected SNPs in additional samples from 1,803 T2D cases and 1,473 controls. The controls were randomly selected from the Taiwan Han Chinese Cell and Genome Bank [94]. The criteria for controls in the association study were (1) no past diagnostic history of T2D, (2) HbA_1C_ ranging from 3.4 to 6, and (3) BMI<32. The two control groups were comparable with respect to BMI, gender, age at study, and level of HbA_1C_. All of the participating T2D cases and controls were of Han Chinese origin, which is the origin of 98% of the Taiwan population. Details of demographic data are shown in Table S10.

### Genotyping

Genomic DNA was extracted from peripheral blood using the Puregene DNA isolation kit (Gentra Systems, Minneapolis, MN, USA). In stage 1, whole genome genotyping using the Illumina HumanHap550-Duo BeadChip was performed by deCODE Genetics (Reykjavík, Iceland). Genotype calling was performed using the standard procedure implemented in BeadStudio (Illumina, Inc., San Diego, CA, USA), with the default parameters suggested by the platform manufacturer. Quality control of genotype data was performed by examining several summary statistics. First, the ratio of loci with heterozygous calls on the X chromosome was calculated to double-check the subject's gender. Total successful call rate and the minor allele frequency of cases and controls were also calculated for each SNP. SNPs were excluded if they: (1) were nonpolymorphic in both cases and controls, (2) had a total call rate <95% in the cases and controls combined, (3) had a minor allele frequency <5% and a total call rate <99% in the cases and controls combined, and (4) had significant distortion from Hardy–Weinberg equilibrium in the controls (*P*<10^−7^). Genotyping validation was performed using the Sequenom iPLEX assay (Sequenom MassARRAY system; Sequenom, San Diego, CA, USA). In the replication stage (stage 2), SNPs showing significant or suggestive associations with T2D and their neighboring SNPs within the same LD block were genotyped using the Sequenom iPLEX assay. The neighboring SNPs in the same LD were selected from the HapMap Asian (CHB + JPT) group data for fine mapping the significant signal.

### Statistical analysis

**Figure 3:**
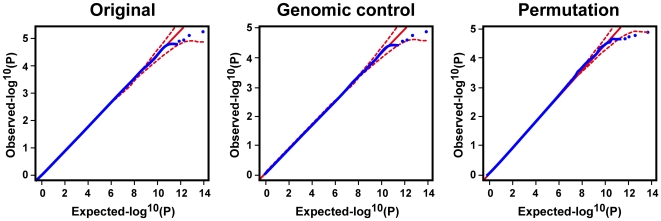
Q–Q plot for the trend test. Q–Q plots are shown for the trend test based on the 516,212 quality SNPs of the initial analysis of 995 cases and 894 controls. The red lines represent the upper and lower boundaries of the 95% confidence bands.

T2D association analysis was carried out to compare allele frequency and genotype distribution between cases and controls using five single-point methods for each SNP: genotype, allele, trend (Cochran–Armitage test), dominant, and recessive models. The most significant test statistic obtained from the five models was chosen. SNPs with *P* values less than a = 2×10^−8^, a cut-off for the multiple comparison adjusted by Bonferroni correction, were considered to be significantly associated with the traits. The joint analysis was conducted by combining the data from the stage 1 and 2 samples. We also applied Fisher's method to combine *P* values for joint analysis. The permutation test was carried out genome-wide for 10^6^ permutations, in which the phenotypes of subjects were randomly rearranged. For better estimation of empirical *P* values, the top SNPs were reexamined using 10^8^ permutations. Each permutation proceeded as follows: (1) the case and control labels were shuffled and redistributed to subjects, and (2) the test statistics of the corresponding association test was calculated based on the shuffled labels. The empirical *P* value was defined as the number of permutations that were at least as extreme as the original divided by the total number of permutations. Detection of possible population stratification that might influence association analysis was carried out using principle component analysis, multidimensional scaling analysis, and genomic control (Text S1). Quantile–quantile (Q–Q) plots were then used to examine *P* value distributions (Figure 3 and Figure S5).

## Supporting Information

Figure S1 Principle component analysis (PCA) plot. The PCA plot shows the first two principal components, estimated by EIGENSTRAT (Price et al. Nat Genet 38: 904–909), based on genotype data from 76,673 SNPs with equal spacing across the human genome. No population stratification between the 995 T2D cases (green x) and 894 controls (red +) was detected (*P* = 0.111, and Fst statistics between populations <0.001).
(1.18 MB TIF)

Figure S2 Multidimensional scaling analysis (MDS) plot. The MDS plot shows the first two principal components, estimated by PLINK (Zheng et al. Am J Hum Genet 81:559–575), based on genotype data from 516,212 SNPs. No population stratification between the 995 T2D cases (red) and 894 controls (blue) was detected (IBS group-difference empirical *P* = 0.192598 for T1: case/control less similar).
(0.74 MB TIF)

Figure S3 LD block between rs231361 and rs223787.
(0.17 MB TIF)

Figure S4 Comparisons to susceptible regions reported by previous GWAS. For each of the (A) *NOTCH2*, (B) *THADA*, (C) *PPARG*, (D) *IGF2BP2*, (E) *ADAMTS9*, (F) *WFS1*, (G) *CDKAL1*, (H) *JAF1*, (I) *SLC30A8*, (J) *CDKN2AB*, (K) *HHEX*, (L) *CDC123/CAMK1D*, (M) *TCF7L2*, (N) *KCNJ11*, (O) *MTNR1B*, (P) *TSPAN8/LGR5*, (Q) *FTO*, and (R) *TCF* (*HNF1B*) regions, the −log_10_ *P* values from the primary scan are plotted as a function of genomic position (NCBI Build 36). The reported SNPs in previous GWAS are denoted by blue diamonds. Estimated recombination rates (right y-axis) based on the Chinese HapMap population are plotted to reflect the local LD structure around the significant SNPs. Gene annotations and numbers of transcripts were taken from NCBI.
(4.17 MB TIF)

Figure S5 Quantile-quantile (QQ) plots. QQ plots are shown for the four association tests, (A) allelic, (B) genotype, (C) dominant, and (D) recessive, based on the 516,212 quality SNPs of the initial analysis of 995 cases and 894 controls. The upper and lower boundaries of the 95% confidence bands are represented by the red lines.
(3.10 MB TIF)

Table S1 Quality control of the subject participants in stage 1.
(0.03 MB DOC)

Table S2 Quality control of the genotyping results.
(0.03 MB DOC)

Table S3 Association results in stage 1.
(0.05 MB DOC)

Table S4 Concordance rates for the 10 SNPs with significant associations in stage 1.
(0.05 MB DOC)

Table S5 Power Calculation using CaTS.
(0.05 MB DOC)

Table S6 Association of additional SNPs within KCNQ1 in all T2D cases and controls in the joint analysis.
(0.06 MB DOC)

Table S7 Conditional analysis on rs2237895.
(0.03 MB DOC)

Table S8 Previously reported loci and SNPs associated with T2D.
(0.13 MB DOC)

Table S9 Genotype frequency and allele frequency of rs17584499 (founders only) from HapMap3.
(0.05 MB DOC)

Table S10 Clinical characteristics of the subjects.
(0.04 MB DOC)

Text S1 Supplementary methods.
(0.03 MB DOC)
