# Supplementary figures and images for: A Genome-Wide Association Study Identifies Susceptibility Variants for Type 2 Diabetes in Han Chinese
Source: PLoS Genet. 2010 Feb 19;6(2):e1000847. doi: 10.1371/journal.pgen.1000847 (PMC2824763; doi:10.1371/journal.pgen.1000847)

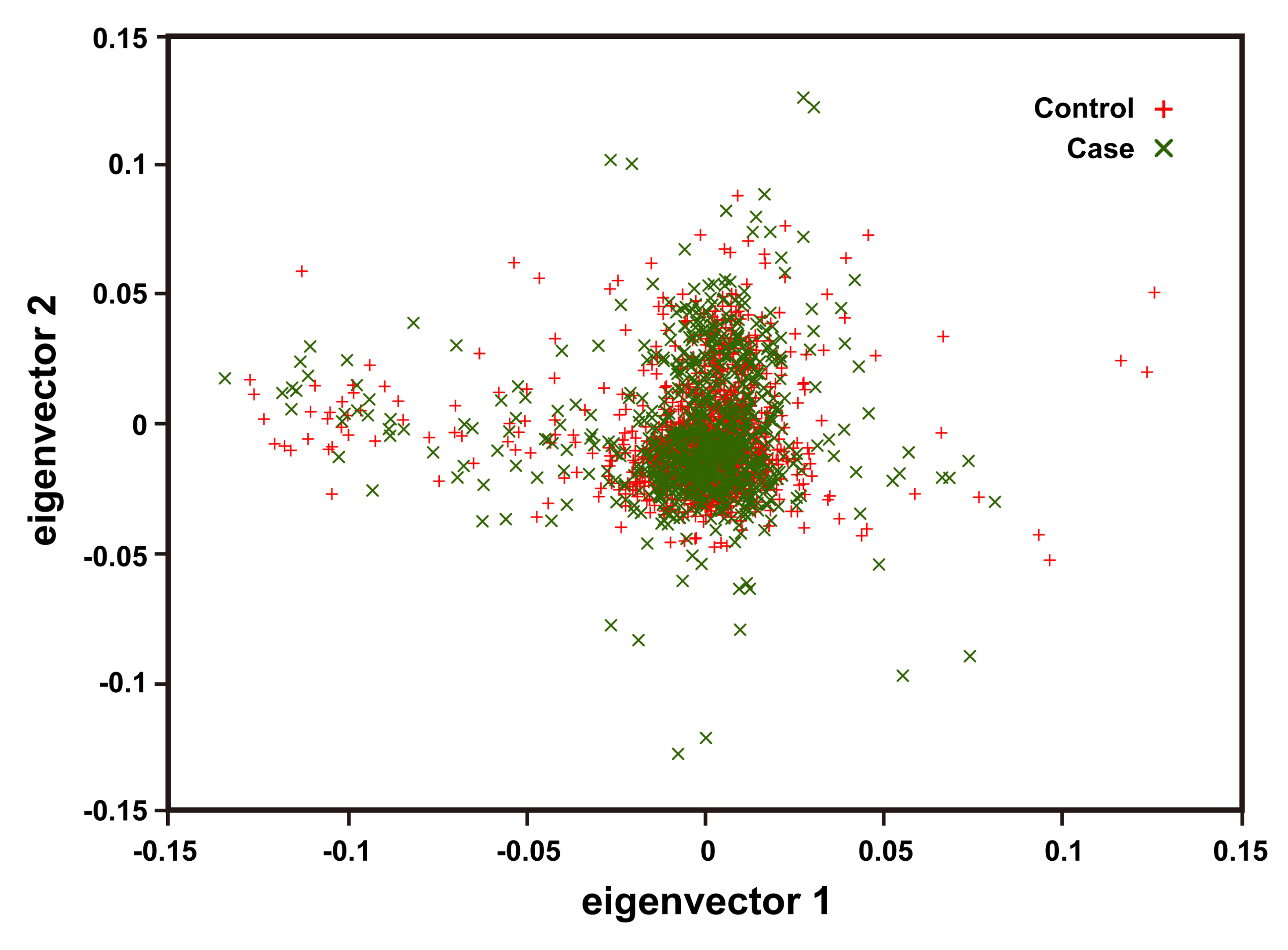

Supplement: Figure S1 — Principle component analysis (PCA) plot. The PCA plot shows the first two principal components, estimated by EIGENSTRAT (Price et al. Nat Genet 38: 904–909), based on genotype data from 76,673 SNPs with equal spacing across the human genome. No population stratification between the 995 T2D cases (green x) and 894 controls (red +) was detected (P = 0.111, and Fst statistics between populations <0.001). (1.18 MB TIF) [file pgen.1000847.s001.tif]

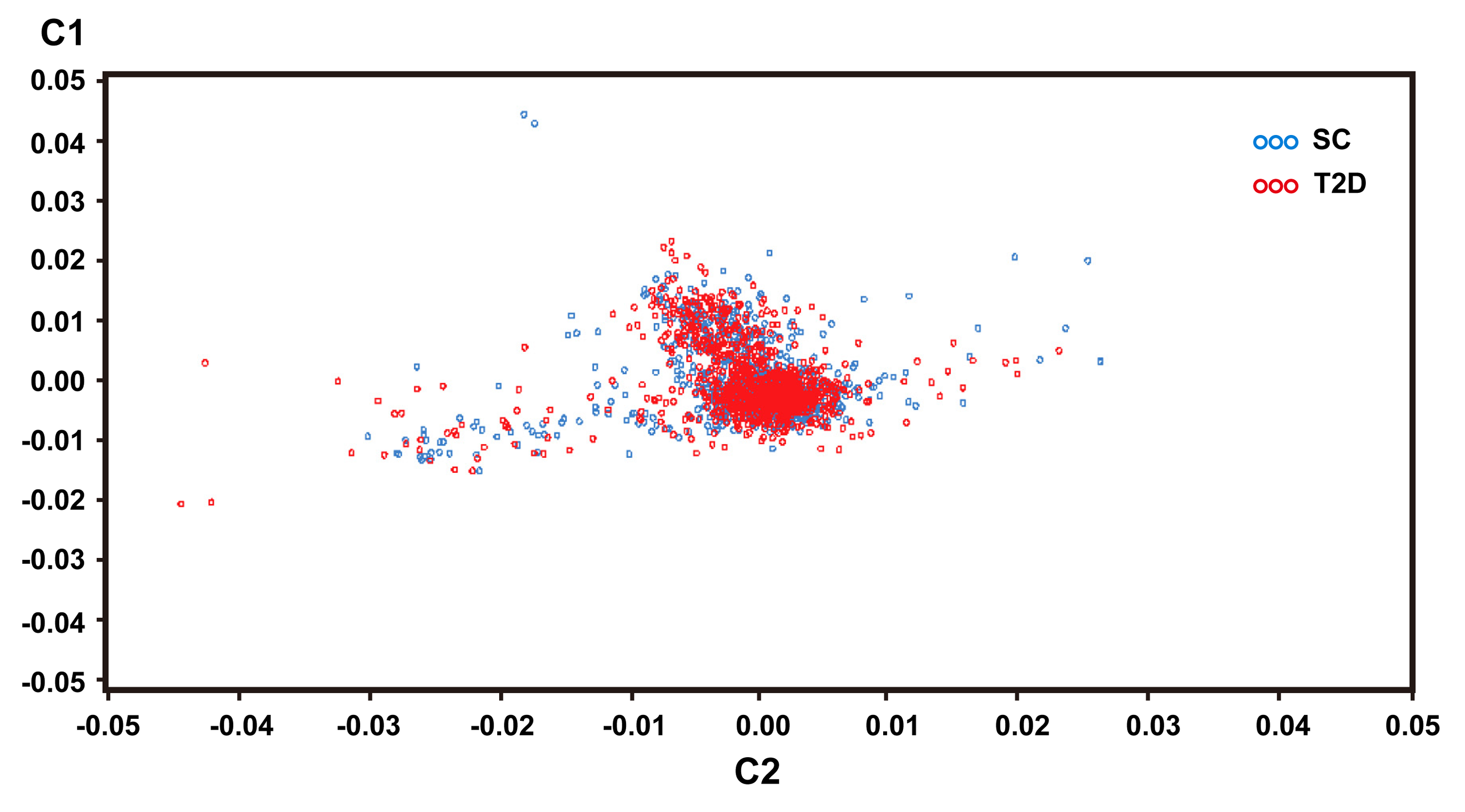

Supplement: Figure S2 — Multidimensional scaling analysis (MDS) plot. The MDS plot shows the first two principal components, estimated by PLINK (Zheng et al. Am J Hum Genet 81:559–575), based on genotype data from 516,212 SNPs. No population stratification between the 995 T2D cases (red) and 894 controls (blue) was detected (IBS group-difference empirical P = 0.192598 for T1: case/control less similar). (0.74 MB TIF) [file pgen.1000847.s002.tif]

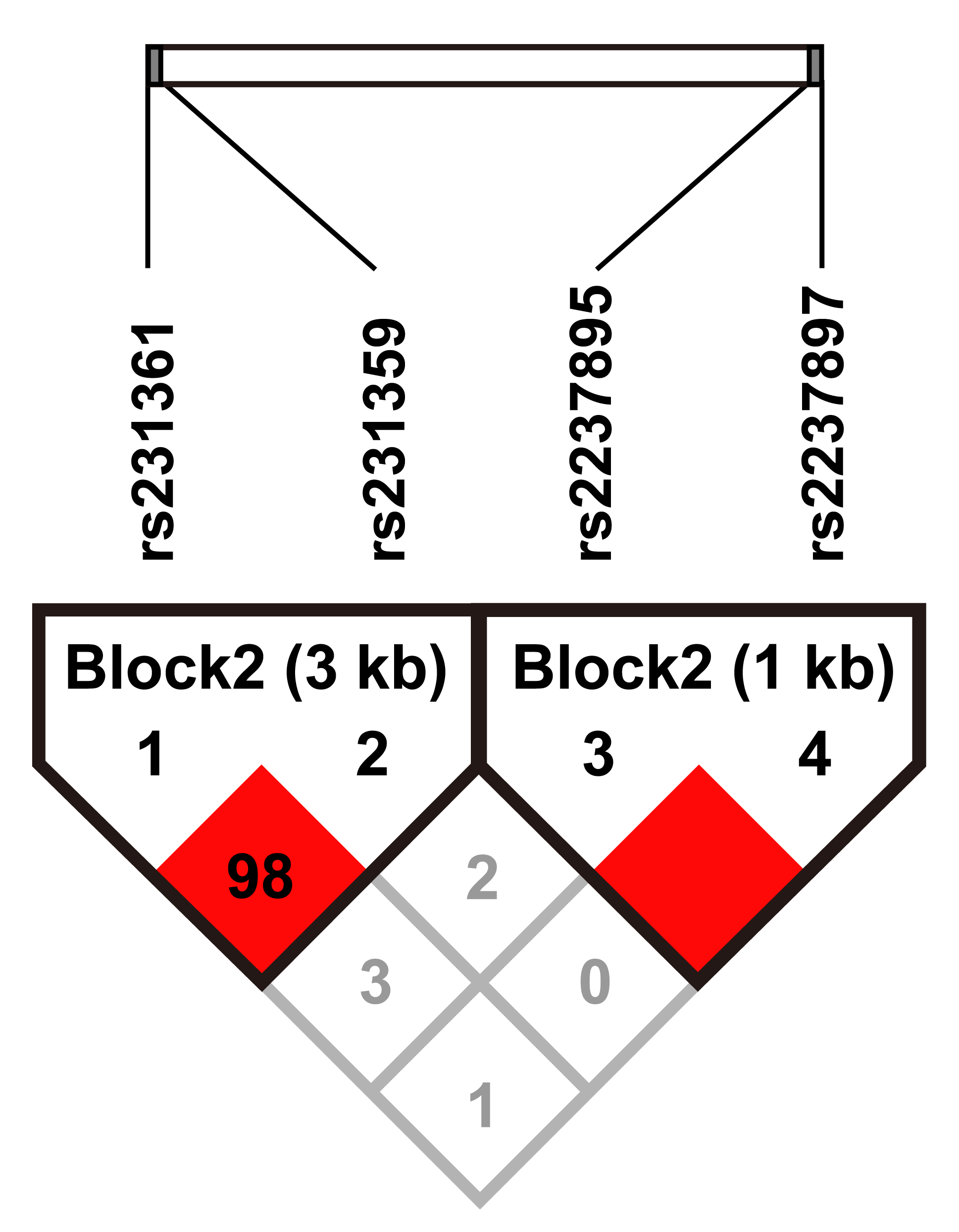

Supplement: Figure S3 — LD block between rs231361 and rs223787. (0.17 MB TIF) [file pgen.1000847.s003.tif]

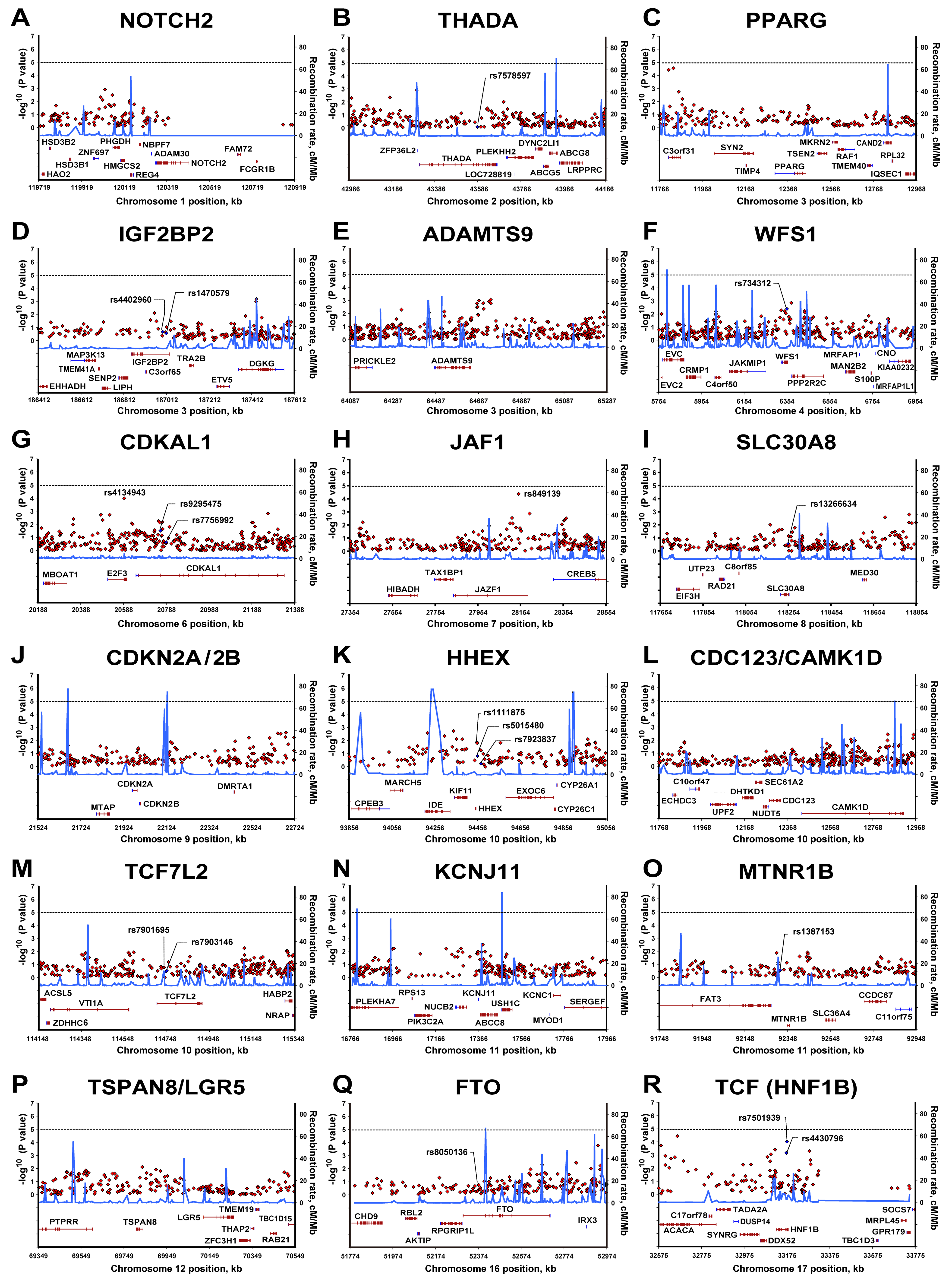

Supplement: Figure S4 — Comparisons to susceptible regions reported by previous GWAS. For each of the (A) NOTCH2, (B) THADA, (C) PPARG, (D) IGF2BP2, (E) ADAMTS9, (F) WFS1, (G) CDKAL1, (H) JAF1, (I) SLC30A8, (J) CDKN2AB, (K) HHEX, (L) CDC123/CAMK1D, (M) TCF7L2, (N) KCNJ11, (O) MTNR1B, (P) TSPAN8/LGR5, (Q) FTO, and (R) TCF (HNF1B) regions, the −log10 P values from the primary scan are plotted as a function of genomic position (NCBI Build 36). The reported SNPs in previous GWAS are denoted by blue diamonds. Estimated recombination rates (right y-axis) based on the Chinese HapMap population are plotted to reflect the local LD structure around the significant SNPs. Gene annotations and numbers of transcripts were taken from NCBI. (4.17 MB TIF) [file pgen.1000847.s004.tif]

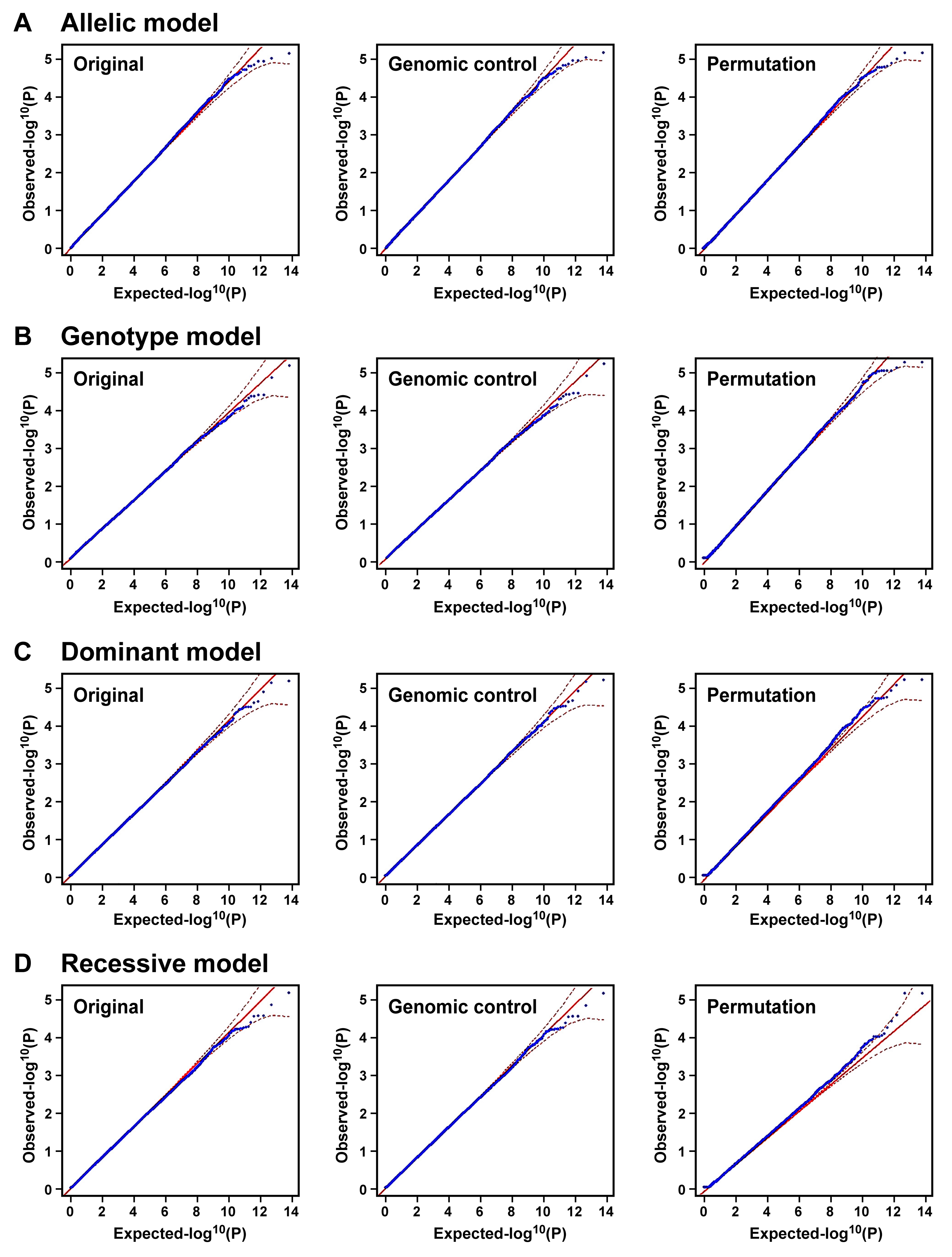

Supplement: Figure S5 — Quantile-quantile (QQ) plots. QQ plots are shown for the four association tests, (A) allelic, (B) genotype, (C) dominant, and (D) recessive, based on the 516,212 quality SNPs of the initial analysis of 995 cases and 894 controls. The upper and lower boundaries of the 95% confidence bands are represented by the red lines. (3.10 MB TIF) [file pgen.1000847.s005.tif]
