# Supplementary material for: A Genome-Wide Association Study Identifies Susceptibility Variants for Type 2 Diabetes in Han Chinese
Source: PLoS Genet. 2010 Feb 19;6(2):e1000847. doi: 10.1371/journal.pgen.1000847 (PMC2824763; doi:10.1371/journal.pgen.1000847)
Supplement: Table S1 — Quality control of the subject participants in stage 1. (0.03 MB DOC) [file pgen.1000847.s006.doc]

**Table S1. Quality control of the subject participants in stage 1.**

|  | | Controls | Cases |
| --- | --- | --- | --- |
| Number at start of QC | | 1,000 | 999 |
| Number dropped during exclusion step: | |  |  |
|  | duplicate | 0 | 0 |
|  | overall call rate <0.95 | 0 | 0 |
|  | controls with HbA1C <3.4 or >6 | 86 | — |
|  | controls with medical record as T2D patients | 51 | — |
|  | sex or BMI missing | 7 | 4 |
| Number at end of QC | | 894 | 995 |
