# Supplementary material for: A Genome-Wide Association Study Identifies Susceptibility Variants for Type 2 Diabetes in Han Chinese
Source: PLoS Genet. 2010 Feb 19;6(2):e1000847. doi: 10.1371/journal.pgen.1000847 (PMC2824763; doi:10.1371/journal.pgen.1000847)
Supplement: Table S2 — Quality control of the genotyping results. (0.03 MB DOC) [file pgen.1000847.s007.doc]

**Table S2. Quality control of the genotyping results.**

|  | | Genome scan | Replication stage | additional genotyping |
| --- | --- | --- | --- | --- |
|  |  | (Stage 1, SNPs) | (Stage 2, SNPs) | within KCNQ1 (SNPs) |
| Number at start of QC | | 560,184 | 39 | 17 |
| Number dropped during exclusion step: | |  |  |  |
|  | SNPs that were nonpolymorphic in case and control | 30,006 | 1 | 0 |
|  | SNPs with overall call rate <0.95 | 211 | 0 | 4 |
|  | SNPs with overall MAF<0.05 and overall call rate <0.99 | 693 | 1 | 1 |
|  | SNPs with HWE for control (*P* < 10^−4^) and no nonpolymorphic in control | 13,456 | 2 | 3 |
| Number at end of QC | | 516,212 | 35 | 13 |
