# Supplementary material for: A Genome-Wide Association Study Identifies Susceptibility Variants for Type 2 Diabetes in Han Chinese
Source: PLoS Genet. 2010 Feb 19;6(2):e1000847. doi: 10.1371/journal.pgen.1000847 (PMC2824763; doi:10.1371/journal.pgen.1000847)
Supplement: Table S3 — Association results in stage 1. (0.05 MB DOC) [file pgen.1000847.s008.doc]

**Table S3. Association results in stage 1.**

|  |  | *P* _best_ |  | *P* _trend_ | *P*_Permutation_ | *P*_PCA-correction_ | *P*_Genomic Control_ |
| --- | --- | --- | --- | --- | --- | --- | --- |
| Chr | SNP |  |  |  | (trend) | ( additive) | ( trend) |
| 4 | rs9985652 | 3.87x10^-6^ | (dom) | 9.30x10^-4^ | 6.62x10^-4^ | 0.001 | 0.001 |
| 4 | rs2044844 | 3.87x10^-6^ | (rec) | 9.30x10^-4^ | 8.72x10^-4^ | 0.002 | 0.001 |
| 16 | rs7192960 | 4.29x10^-6^ | (rec) | 7.66x10^-6^ | 9.98x10^-6^ | 1.35x10^-5^ | 1.64x10^-5^ |
| 20 | rs7361808 | 4.36x10^-6^ | (geno) | 2.30x10^-4^ | 2.54x10^-4^ | 1.55x10^-4^ | 3.89x10^-4^ |
| 10 | rs1751960 | 9.56x10^-6^ | (allele) | 1.13x10^-5^ | 6.65x10^-6^ | 2.87x10^-6^ | 2.35x10^-5^ |
| 1 | rs4845624 | 5.85x10^-6^ | (trend) | 5.85x10^-6^ | 5.85x10^-6^ | 1.18x10^-5^ | 1.27x10^-5^ |
| 17 | rs391300 | 7.61x10^-6^ | (dom) | 9.00x10^-5^ | 7.90x10^-5^ | 5.68x10^-5^ | 1.62x10^-4^ |
| 17 | rs4523957 | 3.24x10^-4^ | (trend) | 3.24x10^-4^ | 3.47x10^-4^ | 2.06x10^-4^ | 5.35x10^-4^ |
| 13 | rs648538 | 8.28x10^-6^ | (rec) | 2.92x10^-4^ | 4.06x10^-4^ | 6.19x10^-4^ | 4.86x10^-4^ |
| 9 | rs17584499 | 2.36x10^-5^ | (rec) | 1.41x10^-4^ | 1.03x10^-4^ | 3.50x10^-4^ | 2.47x10^-4^ |
| 11 | rs231361 | 1.27x10^-4^ | (allele) | 1.49x10^-4^ | 1.93x10^-4^ | 2.46x10^-4^ | 2.59x10^-4^ |
| 11 | rs231359 | 4.20x10^-4^ | (allele) | 4.56x10^-4^ | 6.97x10^-4^ | 7.40x10^-4^ | 9.06x10^-4^ |
| 11 | rs2237895 | 2.92x10^-4^ | (trend) | 2.92x10^-4^ | 3.45x10^-4^ | 0.001 | 4.86x10^-4^ |
