# Supplementary material for: A Genome-Wide Association Study Identifies Susceptibility Variants for Type 2 Diabetes in Han Chinese
Source: PLoS Genet. 2010 Feb 19;6(2):e1000847. doi: 10.1371/journal.pgen.1000847 (PMC2824763; doi:10.1371/journal.pgen.1000847)
Supplement: Table S4 — Concordance rates for the 10 SNPs with significant associations in stage 1. (0.05 MB DOC) [file pgen.1000847.s009.doc]

**Table S4. Concordance rates for the 10 SNPs with significant associations in the stage 1.**

|  |  |  |  | Illumina 550K | |  | Sequenom MassARRAY | |  |
| --- | --- | --- | --- | --- | --- | --- | --- | --- | --- |
|  |  |  |  | Call rate | |  | Call rate | |  |
| SNPs | Chromosome | Position |  | Case | Control |  | Case | Control | Concordance rate |
| rs4845624 | 1 | 150094437 |  | 1 | 1 |  | 1 | 0.995 | 0.998 |
| rs2044844 | 4 | 42551396 |  | 1 | 1 |  | 0.986 | 0.989 | 0.985 |
| rs9985652 | 4 | 42553210 |  | 1 | 1 |  | 0.994 | 0.992 | 0.995 |
| rs17584499 | 9 | 8869118 |  | 1 | 1 |  | 1 | 0.995 | 0.997 |
| rs1751960 | 10 | 29675862 |  | 0.998 | 1 |  | 0.995 | 0.992 | 0.994 |
| rs231361 | 11 | 2648076 |  | 1 | 1 |  | 1 | 0.995 | 0.986 |
| rs648538 | 13 | 29710309 |  | 1 | 1 |  | 1 | 0.995 | 0.997 |
| rs7192960 | 16 | 77974064 |  | 1 | 1 |  | 1 | 0.995 | 0.971 |
| rs391300 | 17 | 2163008 |  | 1 | 1 |  | 1 | 0.995 | 0.995 |
| rs7361808 | 20 | 1784935 |  | 1 | 1 |  | 1 | 0.995 | 0.992 |
