# Supplementary material for: A Genome-Wide Association Study Identifies Susceptibility Variants for Type 2 Diabetes in Han Chinese
Source: PLoS Genet. 2010 Feb 19;6(2):e1000847. doi: 10.1371/journal.pgen.1000847 (PMC2824763; doi:10.1371/journal.pgen.1000847)
Supplement: Table S5 — Power Calculation using CaTS. (0.05 MB DOC) [file pgen.1000847.s010.doc]

**Table S5. Power Calculation using CaTS****

| Disease |  |  |  | Power (%) |  |
| --- | --- | --- | --- | --- | --- |
| T2D | Disease Allele Freq. | Genotype Relative Risk | One Stage Design | Replication Analysis | Joint Analysis |
| (prevalence 6%) | 0.01 | 1.2 | 0 | 0 | 0 |
|  |  | 1.5 | 0 | 0 | 0 |
|  |  | 1.8 | 5 | 2 | 3 |
|  |  | 2 | 18 | 9 | 9 |
|  |  | 2.5 | 81 | 49 | 49 |
|  |  | 3 | 99 | 85 | 85 |
|  | 0.05 | 1.2 | 0 | 0 | 0 |
|  |  | 1.5 | 43 | 22 | 23 |
|  |  | 1.8 | 99 | 83 | 83 |
|  |  | 2 | 100 | 97 | 97 |
|  | 0.1 | 1.2 | 1 | 1 | 1 |
|  |  | 1.5 | 94 | 66 | 66 |
|  |  | 1.8 | 100 | 99 | 99 |
|  |  | 2 | 100 | 100 | 100 |
|  | 0.15 | 1.2 | 4 | 2 | 2 |
|  |  | 1.5 | 99 | 85 | 85 |
|  |  | 1.8 | 100 | 100 | 100 |
|  |  | 2 | 100 | 100 | 100 |

in stage 1 : 995 cases vs 894 controls; in stage 2 : 1803 cases vs 1473 controls

** Skal et al. *Nat Genet* (2006) **38**:209-213.
