# Supplementary material for: A Genome-Wide Association Study Identifies Susceptibility Variants for Type 2 Diabetes in Han Chinese
Source: PLoS Genet. 2010 Feb 19;6(2):e1000847. doi: 10.1371/journal.pgen.1000847 (PMC2824763; doi:10.1371/journal.pgen.1000847)
Supplement: Table S6 — Association of additional SNPs within KCNQ1 in all T2D cases and controls in the joint analysis. (0.06 MB DOC) [file pgen.1000847.s011.doc]

**Table S6. Association of additional SNPs within KCNQ1 in all T2D cases and controls in the joint analysis.**

|  |  |  |  |  |  |  |  |  | *P* value |  |  |
| --- | --- | --- | --- | --- | --- | --- | --- | --- | --- | --- | --- |
| No. | dbSNP | position | Description | Risk allele | RAF (T2D) | RAF (NC) | genotype | allele | trend | dominant | recessive |
| Previously reported T2D-associated SNPs | | | |  |  |  |  |  |  |  |  |
| 1 | rs163170 | 2778003 | c.1794+22160C>T | T | 0.64 | 0.62 | 0.029 | 0.008 | 0.008 | 0.021 | 0.046 |
| 2 | rs2074196^a^ | 2781376 | c.1794+25533G>T | G | 0.66 | 0.64 | 0.036 | 0.033 | 0.032 | 0.213 | 0.011 |
| 3 | rs2074197 | 2781855 | c.1794+26012C>T | C | 0.66 | 0.64 | 0.016 | 0.014 | 0.014 | 0.132 | 0.005 |
| 4 | rs11602964 | 2782447 | c.1794+26604C>T | C | 0.66 | 0.63 | 0.021 | 0.014 | 0.013 | 0.11 | 0.008 |
| 5 | rs7480855 | 2792661 | c.1794+36818A>G | A | 0.66 | 0.63 | 0.007 | 0.001 | 0.002 | 0.005 | 0.021 |
| 6 | rs163177 | 2794989 | c.1794+39146T>C | C | 0.5 | 0.44 | 1.5x10^-7^ | 4.7x10^-8^ | 5.3x10^-8^ | 1.4x10^-7^ | 3.7x10^-4^ |
| 7 | rs163182 | 2800792 | c.1794+44949G>C | C | 0.41 | 0.37 | 6.8x10^-5^ | 1.5x10^-5^ | 1.5x10^-5^ | 4.1x10^-5^ | 0.005 |
| 8 | rs2283228^b^ | 2806106 | c.1794+50263A>C | A | 0.67 | 0.63 | 0.002 | 3.5x10^-4^ | 3.8x10^-4^ | 0.003 | 0.006 |
| 9 | rs2237895^a,b^ | 2813770 | c.1794+57927A>C | C | 0.09 | 0.05 | 6.5x10^-9^ | 1.2x10^-9^ | 9.7x10^-10^ | 1.7x10^-8^ | 4.9x10^-5^ |
| 10 | rs2237897^b^ | 2815122 | c.1794+59279C>T | C | 0.7 | 0.65 | 3.6x10^-7^ | 1.4x10^-7^ | 1.6x10^-7^ | 4.9x10^-5^ | 2.5x10^-6^ |
| Selected from sequencing results of KCNQ1 | | | |  |  |  |  |  |  |  |  |
| 11 | rs12720449 | 2566610 | c.1343C>G (P448R) | C | 0.89 | 0.88 | 0.338 | 0.144 | 0.142 | 0.145 | 0.602 |
| 12 | New | 2825444 | c.1795-129G>A | A | 0.06 | 0.05 | 0.31 | 0.167 | 0.167 | 0.14 | 0.882 |
| 13 | New | 2826646 | c.*837G>A | G | 0.96 | 0.95 | 0.828 | 0.944 | 0.944 | 0.992 | 0.546 |

^a^Two most significant SNPs reported by Yasuda et al. (Nat Genet [2008] 40:1092-1097).

^b^Three most significant SNPs reported by Unoki et al. (Nat Genet [2008] 40:1098-1102.

RAF (T2D) and RAF (NC): risk allele frequencies in cases and controls, respectively.
