# Supplementary material for: A Genome-Wide Association Study Identifies Susceptibility Variants for Type 2 Diabetes in Han Chinese
Source: PLoS Genet. 2010 Feb 19;6(2):e1000847. doi: 10.1371/journal.pgen.1000847 (PMC2824763; doi:10.1371/journal.pgen.1000847)
Supplement: Table S7 — Conditional analysis on rs2237895. (0.03 MB DOC) [file pgen.1000847.s012.doc]

**Table S7. Conditional analysis on rs2237895.**

| Parameter | *P* value | OR | 95% CI |
| --- | --- | --- | --- |
| rs231361 | 0.0774 | 0.476 | 0.209–1.085 |
| rs231359 | 0.3247 | 1.517 | 0.662–3.474 |
