# Supplementary material for: A Genome-Wide Association Study Identifies Susceptibility Variants for Type 2 Diabetes in Han Chinese
Source: PLoS Genet. 2010 Feb 19;6(2):e1000847. doi: 10.1371/journal.pgen.1000847 (PMC2824763; doi:10.1371/journal.pgen.1000847)
Supplement: Table S8 — Previously reported loci and SNPs associated with T2D. (0.13 MB DOC) [file pgen.1000847.s013.doc]

**Table S8. Previously reported loci and SNPs associated with T2D.**

|  |  |  |  |  | Previous GWAS |  |  | This study (stage 1) | |
| --- | --- | --- | --- | --- | --- | --- | --- | --- | --- |
| Genes | Chr | SNP | Position (bp) | Risk/nonrisk allele | *P* value | references |  | Risk/nonrisk allele | *P* value (Trend) |
| PPARG | 3 | rs1801282 | 12368125 | C/G | 1.7 × 10^−6^ | 27,32,33 |  | **—** | **—** |
| KCNJ11 | 11 | rs5219 | 17366148 | T/C | 6.7 × 10^−11^ | 27,32,33 |  | **—** | **—** |
| CDKAL1 | 6 | rs7754840 | 20769229 | C/G | 4.1 × 10^−11^ | 27 |  | **—** | **—** |
|  |  | rs4712524 | 20765844 | G/A | 3.3 × 10^−10^ | 29 |  | **—** | **—** |
|  |  | rs9295475* | 20760744 | G/A | 4.1 × 10^−9^ | 29 |  | G/A | 0.16723 |
|  |  | rs9460546 | 20771611 | G/T | 3.4 × 10^−10^ | 29 |  | **—** | **—** |
|  |  | rs7756992* | 20787688 | G/A | 7.7 × 10^−9^ | 30 |  | G/A | 0.51052 |
|  |  | rs9465871 | 20825234 | C/T | 1.02 × 10^−6^ | 31 |  | **—** | **—** |
|  |  | rs7754840 | 20769229 | C/G | 2.4 × 10^−3^ | 32 |  | **—** | **—** |
|  |  | rs10946398 | 20769013 | C/A | 4.1 × 10^−11^ | 32,33 |  | **—** | **—** |
|  |  | **rs4134943**** | **20591386** | **—** | **—** |  |  | **C/T** | **0.00011** |
| CDKN2A/B | 9 | rs10811661 | 22124094 | T/C | 7.8 × 10^−15^ | 27,32,33 |  | **—** | **—** |
|  |  | rs564398 | 22019547 | — | 1.2 × 10^−7^ | 33 |  | **—** | **—** |
| HHEX | 10 | rs5015480* | 94455539 | C/T | 5.7 × 10^−10^ | 33 |  | C/T | 0.1538 |
|  |  | rs1111875* | 94452862 | C/T | 5.7 × 10^−10^ (27, 32, 33), 3 × 10^−6^ (38) | 27,32,33,38 |  | C/T | 0.01353 |
|  |  | rs7923837* | 94471897 | G/A | 7.5 × 10^−6^ | 38 |  | G/A | 0.66093 |
| IGF2BP2 | 3 | rs4402960* | 186994381 | T/G | 8.9 × 10^−16^ | 27,32,33 |  | T/G | 0.21805 |
|  |  | rs4376068 | 186980329 | C/A | 2.1 × 10^−9^ | 29 |  | **—** | **—** |
|  |  | rs1470579* | 187011774 | C/A | 1.3 × 10^−9^ | 32 |  | C/A | 0.32837 |
|  |  | rs6769511 | 187012984 | C/T | 1.3 × 10^−9^ | 29 |  | **—** | **—** |
| SLC30A8 | 8 | rs13266634* | 118253964 | C/T | 5.3 × 10^−8^ (27,32,33), 6.1 × 10^−8^ (38) | 27,32,33,38 |  | C/T | 0.28813 |
| FTO | 16 | rs8050136* | 52373776 | A/C | 1.3 × 10^−12^ | 27, 33 |  | A/C | 0.19621 |
|  |  | rs9939609 | 52378028 | A/T | 5.24 × 10^−8^ | 31 |  | **—** | **—** |
| TCF7L2 | 10 | rs7903146* | 114748339 | T/C | 1.0 × 10^−48^ (27, 32), 1.5 × 10^−34^ (38) | 27, 32, 38 |  | T/C | 0.3208 |
|  |  | rs4506565 | 114746031 | T/A | 5.68 × 10^−13^ | 31 |  | **—** | **—** |
|  |  | rs7901695* | 114744078 | C/T | 1 × 10^−48^ | 33 |  | C/T | 0.36758 |
| JAF1 | 7 | rs864745 | 27953796 | T/C | 5 × 10^−14^ | 34 |  | **—** | **—** |
|  |  | **rs849139**** | **28145921** | **—** | **—** |  |  | **C/T** | **0.00057** |
| CDC123, CAMK1D | 10 | rs12779790 | 12368016 | G/A | 1.2 × 10^−10^ | 34 |  | **—** | **—** |
| TSPAN8, LGR5 | 12 | rs7961581 | 69949369 | C/T | 1.1 × 10^−9^ | 34 |  | **—** | **—** |
| THADA | 2 | rs7578597* | 43586327 | T/C | 1.1 × 10^−9^ | 34 |  | T/C | 0.81945 |
| ADAMTS9 | 3 | rs4607103 | 64686944 | C/T | 1.2 × 10^−8^ | 34 |  | **—** | **—** |
| NOTCH2 | 1 | rs10923931 | 120319482 | T/G | 4.1 × 10^−8^ | 34 |  | **—** | **—** |
| MTNR1B | 11 | rs1387153* | 92313476 | — | 7.6 × 10^−29^ (FPG), 6.3 × 10^−5^ (T2D) | 35 |  | C/T | 0.92231 |
|  |  | rs10830963 | 92348358 | — | 3.2 × 10^−50^ | 37 |  | **—** | **—** |
| TCF (HNF1B) | 17 | rs757210 | 33170628 | — | 5 × 10^−6^ | 25 |  | **—** | **—** |
|  |  | **rs7501939*, **** | **33175269** | C/T | **9.2 × 10^−7^** | **24** |  | **T/C** | **0.00012** |
|  |  | rs4430796* | 33172153 | A/G | 2.7 × 10^−7^ | 24 |  | G/A | 0.00071 |
| WFS1 |  | rs10010131 | 6343816 | — | 1.4 × 10^−7^ | 26 |  | **—** | **—** |
|  |  | rs6446482 | 6346594 | — | 3.4 × 10^−7^ | 26 |  | **—** | **—** |
|  |  | rs734312* | 6354255 | — | 2 × 10^−5^ | 26 |  | A/G | 0.00709 |

*Reported SNPs associated with T2D in previous GWA scans and genotyped in our GWA scan.

**SNPs with a *P* value ranging from 5 × 10^−4^ to 10^−5^ in our GWA scan.
