# Supplementary material for: A Genome-Wide Association Study Identifies Susceptibility Variants for Type 2 Diabetes in Han Chinese
Source: PLoS Genet. 2010 Feb 19;6(2):e1000847. doi: 10.1371/journal.pgen.1000847 (PMC2824763; doi:10.1371/journal.pgen.1000847)
Supplement: Table S9 — Genotype frequency and allele frequency of rs17584499 (founders only) from HapMap3. (0.05 MB DOC) [file pgen.1000847.s014.doc]

**Table S9. Genotype frequency and allele frequency of rs17584499 (founders only) from HapMap3.**

|  | Genotype frequency | | |  | Allele frequency | |  |
| --- | --- | --- | --- | --- | --- | --- | --- |
| Population | N (%) | | |  | % | | Fst |
|  | CC | CT | TT |  | C | T | overall |
| CHB | 69 (82.14) | 15 (17.86) | 0 (0.00) |  | 0.91 | 0.09 |  |
| CHD | 76 (89.41) | 9 (10.59) | 0 (0.00) |  | 0.95 | 0.05 |  |
| JPT | 62 (72.09) | 22 (25.58) | 2 (2.33) |  | 0.85 | 0.15 |  |
| CEU | 67 (59.82) | 39 (34.82) | 6 (5.36) |  | 0.77 | 0.23 |  |
| GIH | 47 (53.41) | 36 (40.91) | 5 (5.68) |  | 0.74 | 0.26 |  |
| MEX | 28 (56.00) | 18 (36.00) | 4 (8.00) |  | 0.74 | 0.26 | 0.0688 |
| TSI | 48 (54.55) | 38 (43.18) | 2 (2.27) |  | 0.76 | 0.24 |  |
| ASW | 46 (93.88) | 3 (6.12) | 0 (0.00) |  | 0.97 | 0.03 |  |
| LWK | 81 (90.00) | 9 (10.00) | 0 (0.00) |  | 0.95 | 0.05 |  |
| MKK^*^ | 103 (72.54) | 37 (26.06) | 2 (1.41) |  | 0.86 | 0.14 |  |
| YRI | 107 (94.69) | 6 (5.31) | 0 (0.00) |  | 0.97 | 0.03 |  |

*A subject in the MKK group had a missing genotype and was excluded from this calculation.

CHB: Han Chinese from Beijing, China; CHD: Chinese from metropolitan Denver, Colorado; JPT: Japanese from Tokyo, Japan;CEU: Utah residents with Northern and Western European ancestry from the CEPH collection; GIH: Gujarati Indians in Houston, Texas ; MEX: Mexican ancestry in Los Angeles, California; TSI: Tuscans from Italy; ASW: African ancestry from the Southwest USA; LWK: Luhya in Webuye, Kenya; MKK: Maasai in Kinyawa, Kenya; YRI: Yoruba in Ibadan, Nigeria.
