# Supplementary material for: A Genome-Wide Association Study Identifies Susceptibility Variants for Type 2 Diabetes in Han Chinese
Source: PLoS Genet. 2010 Feb 19;6(2):e1000847. doi: 10.1371/journal.pgen.1000847 (PMC2824763; doi:10.1371/journal.pgen.1000847)
Supplement: Table S10 — Clinical characteristics of the subjects. (0.04 MB DOC) [file pgen.1000847.s015.doc]

**Table S10. Clinical characteristics of the subjects.**

|  | Stage 1 | |  | Stage 2 | |
| --- | --- | --- | --- | --- | --- |
|  | T2D | Controls |  | T2D | Controls |
| Number | 995 | 894 |  | 1,803 | 1,473 |
| Male (%) | 50.5 | 51.5 |  | 52 | 49.6 |
| Age at study (years) | 59.2 ± 10.2 | 49.8 ± 17.9 |  | 60.8 ± 12.4 | 49.0 ± 17.1 |
| age at diagnosis (years) | 50 ± 9.4 | — |  | 50.7 ± 12.0 | — |
| BMI (kg/m^2^) | 25.1 ± 3.8 | 23 ± 3.3 |  | 25.1 ± 3.9 | 23.3 ± 3.3 |
| HbA_1C_ (%) | 7.9 ± 1.5 | 5.2 ± 0.4 |  | 7.6 ± 2.2 | 5.1 ± 0.4 |
| Fasting plasma glucose (mg/dL) | 144.7 ± 43.0 | — |  | 143.9 ± 55.8 | — |
| Diabetic duration (years) | 9.2 ± 7.2 | — |  | 10.1 ± 2.2 | — |
