# Supplementary material for: A Genome-Wide Association Study Identifies Susceptibility Variants for Type 2 Diabetes in Han Chinese
Source: PLoS Genet. 2010 Feb 19;6(2):e1000847. doi: 10.1371/journal.pgen.1000847 (PMC2824763; doi:10.1371/journal.pgen.1000847)
Supplement: Text S1 — Supplementary methods. (0.03 MB DOC) [file pgen.1000847.s016.doc]

**Supplementary Methods**

**1. Principal component analysis using EIGENSTRAT**

To select unlinked SNPs for principal component analysis (PCA), we first lined up the quality SNPs by physical position within each chromosome and chose the (1+ 4i)th SNPs: *i* = 0, 1, 2, …. Furthermore, we retained only one SNP in each linkage disequilibrium (LD) block in cases with two or more SNPs located in the same LD block. The LD blocks were estimated based on the CHB + JPT dataset (hapmap3_r2_b36_fwd) of the International HapMap Project ([www.hapmap.org](http://www.hapmap.org/)), using Haploview with LD blocks defined by Gabriel et al. [1]. The final set comprised 76,673 SNPs.

We performed principal component analysis using EIGENSTRAT [2] using a default setting and obtained 20 principal components for further tests. Detection of population stratification and ethnicity outliers was carried out based on the principal components. For the purposes of comparison, we also included the 20 principal components in logistic regressions as covariates to assess the association between the top SNPs (selected without PC correction) and the affected status.

**2. Analyses based on pair-wise identity-by-state (IBS) distance using Plink**

Pair-wise identity-by-state (IBS) distance based on all 516,212 quality SNPs was used to identify pairs that might be close relatives of one another. Multidimensional scaling analysis and outlier detection was performed based on the pair-wise IBS distance. A permutation test for between-group IBS differences was performed with a fixed 10,000 permutations. For stratification effects between cases and controls, we reported the *P* value of testing whether or not, on average, an individual was less similar to another phenotypically discordant individual than would be expected by chance (denoted as T1 in PLINK). PLINK [3] was used to carry out the analysis.

**3. Genomic control**

Procedures for genomic control were carried out according to the methods of Devlin and Roeder [4] and Zheng et al. [5]. The variance inflation factor lambda was estimated for trend, allele type, dominant, and recessive chi-square tests, based on all 516,212 quality SNPs. *P* values with genomic control were calculated for comparison.

**References**

1. Gabriel SB, Schaffner SF, Nguyen H, Moore JM, Roy J, et al. (2002) The structure of haplotype blocks in the human genome. Science 296: 2225-2229.

2. Price AL, Patterson NJ, Plenge RM, Weinblatt ME, Shadick NA, et al. (2006) Principal components analysis corrects for stratification in genome-wide association studies. Nat Genet 38: 904-909.

3. Purcell S, Neale B, Todd-Brown K, Thomas L, Ferreira MA, et al. (2007) PLINK: a tool set for whole-genome association and population-based linkage analyses. Am J Hum Genet 81: 559-575.

4. Devlin B, Roeder K (1999) Genomic control for association studies. Biometrics 55: 997-1004.

5. Zheng G, Freidlin B, Gastwirth JL (2006) Robust genomic control for association studies. Am J Hum Genet 78: 350-356.
